# Neurological Soft Signs and Their Relationships to Neurocognitive Functions: A Re-Visit with the Structural Equation Modeling Design

**DOI:** 10.1371/journal.pone.0008469

**Published:** 2009-12-24

**Authors:** Raymond C. K. Chan, Ya Wang, Li Wang, Eric Y. H. Chen, Theo C. Manschreck, Zhan-jiang Li, Xin Yu, Qi-yong Gong

**Affiliations:** 1 Neuropsychology and Applied Cognitive Neuroscience Laboratory, Institute of Psychology, Chinese Academy of Sciences, Beijing, China; 2 Key Laboratory of Mental Health, Institute of Psychology, Chinese Academy of Sciences, Beijing, China; 3 Department of Psychiatry, the University of Hong Kong, Hong Kong Special Administrative Region, China; 4 Department of Psychiatry, Harvard Medical School, Boston, Massuchusetts, United States of America; 5 Beijing Anding Hospital, Capital Medical University, Beijing, China; 6 Institute of Mental Health, Peking University, Beijing, China; 7 Huaxi MR Research Centre, Department of Radiology, West China Hospital/West China School of Medicine, Sichuan University, Chengdu, China; RAND Corporation, United States of America

## Abstract

**Background:**

Neurological soft signs and neurocognitive impairments have long been considered important features of schizophrenia. Previous correlational studies have suggested that there is a significant relationship between neurological soft signs and neurocognitive functions. The purpose of the current study was to examine the underlying relationships between these two distinct constructs with structural equation modeling (SEM).

**Methods:**

118 patients with schizophrenia and 160 healthy controls were recruited for the current study. The abridged version of the Cambridge Neurological Inventory (CNI) and a set of neurocognitive function tests were administered to all participants. SEM was then conducted independently in these two samples to examine the relationships between neurological soft signs and neurocognitive functions.

**Results:**

Both the measurement and structural models showed that the models fit well to the data in both patients and healthy controls. The structural equations also showed that there were modest to moderate associations among neurological soft signs, executive attention, verbal memory, and visual memory, while the healthy controls showed more limited associations.

**Conclusions:**

The current findings indicate that motor coordination, sensory integration, and disinhibition contribute to the latent construct of neurological soft signs, whereas the subset of neurocognitive function tests contribute to the latent constructs of executive attention, verbal memory, and visual memory in the present sample. Greater evidence of neurological soft signs is associated with more severe impairment of executive attention and memory functions. Clinical and theoretical implications of the model findings are discussed.

## Introduction

Neurological soft signs are classically referred to as the minor, non-localizable, and objective abnormalities that are considered to reflect disturbances in connections between subcortical and cortical regions or between cortical regions [1]–[3]. Typical signs include impairments in motor coordination, sensory integration, motor sequencing of complex movements and the corresponding disinhibition of associated movements that can be elicited objectively and rated reliably [1], [4]–[7]. Typical soft signs are seen in the majority of individuals with schizophrenia [8]–[10]. The crucial role of neurological soft signs in schizophrenia has been considered as among the “target features” that encompass the idea that both genetic and non-genetic processes lead to maldevelopment in neurocognitive systems [11], [12].

On the other hand, neurocognitive deficits have also been consistently demonstrated in schizophrenia [13], [14]. Previous studies suggest that the two distinct constructs of neurological soft signs and neurocognitive functions are actually capturing similar underlying deficits in schizophrenia [15]–[18]. In the seminal paper and the subsequent update of the studies of neurological signs and neurocognitive function in schizophrenia, Buchanan and colleagues [4], [19] and others [20] further suggested that these two constructs may serve as the endophenotypes for schizophrenia.

However, previous studies of the relationships between neurological soft signs and neurocognitive performances in schizophrenia have been limited by their designs and methodologies. First, all of the previous studies adopted a correlational design [15]–[17], [21]–[23]. Empirical findings suggest that neurological soft signs and neurocognitive deficits appear to reflect overlapping neural substrates, e.g., a simple correlational design might have underestimated the potential relationship between these two constructs [24]. Although Arango et al. [17] used a combination of covariate-controlled stepwise regression analyses and linear discriminant analyses to investigate the neurocognitive performances in patients with schizophrenia, they did not specifically test the latent constructs of neurocognitive functions and neurological soft signs. Mohr et al. [25] also administered a comprehensive neurocognitive function battery to a group of patients with first episode schizophrenia and healthy controls, and adopted a multivariate profile analysis to examine the corresponding group differences. They found that neurological soft signs were correlated with the main clinical features of the illness and showed a consistent correlation with neurocognitive functioning in schizophrenia. However, Mohr et al's study [26] did not test specifically whether these two constructs, i.e., neurocognitive functions and neurological soft signs, accounted for each other.

Moreover, the majority of the findings were limited to western or Caucasian samples. Buchanan and Heinrichs [5], and Gureje [27] reported that healthy African Americans have an increased level of soft signs compared with Caucasians, sensory integration subscale of the Neurological Evaluation Scale (NES) [5] in particular. Chen and Chan [28] also found that there was a trend for Chinese individuals with schizophrenia to have lower scores in the sensory integration subscore of the Cambridge Neurological Inventory (CNI) [6]. These findings tentatively suggest that among the subscales in the NES and CNI, sensory integration may be more vulnerable to ethnic variation. However, it is not clear whether we can generalize these western-based findings to eastern and non-Caucasian samples.

Finally, in line with the former argument, most of these studies have emphasized the relationship between neurological soft signs and neurocognitive functions in schizophrenia, as very little is known about the corresponding relationships in healthy or non-clinical samples. Most recent empirical findings, e.g., Dazzan et al. [29] indicated that higher rates of neurological soft signs were associated with a reduction of inferior frontal gyrus, middle and superior temporal gyrus, and anterior cingulate gyrus in non-clinical volunteers, indicating that specific brain regions are actually serving for the neuroanatomical substrates of neurological soft signs across healthy individuals and schizophrenic patients. Given that neurological soft signs are also demonstrated in healthy, non-clinical samples, and non-psychotic relatives of patients with schizophrenia [2], [30]–[34], and the observable variations of neurocognitive performances between clinical and non-clinical samples, e.g., it is worthwhile to extend such an investigation to non-clinical samples [35].

Taken together, preliminary studies suggest that neurological soft signs can be considered to be the endophenotype for schizophrenia. Endophenotype has been considered to be an internal phenotype that is not obvious to the unaided eyes and can fill the gap between symptoms and the putative genes that actualize the elusive disease processes of schizophrenia and other psychiatric disorders [36]. Most of the previous studies of endophenotypes have focused primarily on conventional neurocognitive functions such as attention, memory and executive functions. Very little attention has been paid on the study of neurological soft signs as the alternate “neurocognitive” endophenotype for schizophrenia. Given that the administration of conventional neurocognitive function tests is relatively time-consuming, the assessment of a shorter equivalent form of neurocognitive function may save time for the tight clinical routines for practitioners. The nature and characteristics of the neurological soft signs testing suggest that this test can be more feasible for clinicians to screen for any neurocognitive impairment in schizophrenia. The administration of neurological soft signs only takes up about 15 minutes. For instance, the Lurian Fist-Edge-Palm test [37] of motor function is often used in neuropsychological assessment as a quick and easy-to-administer test that is sensitive to schizophrenia [38]. Modifications of this task and other similar movement involving rhythm and repetitive action like simpler finger tapping, alternate finger tapping as well as diadochokinesia have been incorporated as subtests in standardized tests for frontal-executive functions [39]–[41]. To be the potential endophenotypes, the relationship observed in clinical group should also be extended to a non-clinical group, but in an attenuated form, i.e., criterion of association of the illness in population. However, very few studies on neurological soft signs have been equally attended to the non-clinical samples.

The present study adopted the structured equation modeling (SEM) strategy to examine the underlying relationships between neurological soft signs and neurocognitive functions in both healthy volunteers and patients with schizophrenia. The strength of the SEM approach is to examine the latent structure of the two constructs of neurological soft signs and neurocognitive functions and their corresponding relationships simultaneously (for details, refer to the methodology section below). In this way, we can investigate these two constructs at an explanatory level rather than an exploratory level (as the majority of the previous studies have). Given the above arguments, we hypothesized that conventional neurocognitive functions and neurological soft signs serve as the same level of neural basis of higher cortical functioning. Moreover, it was also hypothesized that there were similar associations between neurocognitive measures and neurological soft signs in both schizophrenia and healthy controls; however, such associations may be attenuated in healthy controls.

## Methods

### Participants

The participants included 118 in-patients with schizophrenia (100 men and 18 women) recruited from Queen Mary Hospital of Hong Kong, Institute for Mental Health of the Peking University, and Anding Hospital in Beijing. Part of the findings of the prevalence rate of neurological soft signs in the Hong Kong sample (77 patients) has been reported in our previous study [34], [38]. All of the patients met the DSM-IV [42] criteria for schizophrenia and were outpatients. A consensus diagnosis was arrived at after two experienced psychiatrists performed face-to-face interviews using the Structured Clinical Interview for the DSM-IV. The exclusion criteria included physical illness that involved the central nervous system, life time substance or alcohol abuse and clinical evidence of mental retardation, and comorbid mental illnesses. The mean age and years of education were 40.23 years (SD = 12) and 9.47 years (SD = 3.76), respectively. The mean length of illness was 16.04 years (SD = 11.91).

Another sample of 160 healthy people (62 men and 98 women) was recruited from Beijing and Guangzhou, China. They were all recruited from the sample pool for an assessment of neuropsychological performance. All participants were screened by a brief mental health status questionnaire by research assistants who were trained to administer the questionnaire. This questionnaire was mainly used to capture items whether the healthy volunteers had a family history of mental illnesses based on a dichotomous response. The exclusion criteria were the same as the patient group in addition to having a previous history of mental illnesses. The mean age and mean number of education of the healthy controls were 25.87 years (SD = 8.76) and 14.68 years (SD = 2.56), respectively. The mean IQ estimate assessed by the Wechsler Adult Intelligence Scale – Revised (Chinese version [43], was 117.39 (SD = 18.15). The IQ of these healthy volunteers was relatively high, possibly because these participants were recruited mainly from the local universities of Beijing and Guangzhou. All the patients and healthy volunteers were Han Chinese. Significant differences were found between the patient and healthy groups in terms of age, education, and gender proportion. However, as the main purpose of the study was not to compare the neurocognitive performances between the two groups, such differences would not affect our final modeling results.

### Neurocognitive Functions

Executive attention is evaluated with a set of tests specifically designed to capture the executive component of attention control. The Sustained Attention to Response Task (SART) [44] is a computer test during which participants respond with a key press to the occurrence of a target stimulus (digit) while inhibiting/withholding the response to the non-target digit “3”. This test has been demonstrated to capture sustained attention and disinhibition in clinical samples including schizophrenia [45]. The modified version of the Wisconsin Card Sorting Test (MCST) [46] was used to assess switching and flexibility. The main difference between this version and original version of Wisconsin Card Sorting Test [47] is that the modified version informs the subject about the change of rule of sorting criteria. The Verbal Fluency Test was also used to measure executive function. Participants were instructed to generate as many exemplars of animal names as possible within 1 minute.

Verbal memory and visual memory were measured by the Logical Memory Subscale and Visual Reproduction Subscale from the Chinese version of the Wechsler Memory Scale-Revised [48], respectively.

### Neurological Soft Signs

Neurological soft signs were evaluated with the soft signs subscales of the CNI [6]. The motor coordination subscale consists of items assessing rapid motor movements such as finger-thumb opposition, diadochokinesia, and fist-edge-palm. The sensory integration subscale consists of items evaluating tactile sensation such as extinction, left-right discrimination and stereognosis. The disinhibition subscale consists of items for withholding or inhibiting associated movements such as head movement while performing saccadic tracking of objects and corresponding associated movements (mirror movement) while performing rapid alternating movements in diadochokinesia. In the original scale, scoring was made according to standardised anchor points to indicate “normal” response (scored as 0), “equivocal response” (0.5), “abnormal” response (1) or “grossly abnormal” response (2). In the present study, item scores were dichotomized into either “absent” (covering normal or equivocal) or “present” (covering abnormal or grossly abnormal). Interrater reliability on the subscale scores were calculated for each of the subscales based on investigators' ratings on 15 independent cases by three raters. The intraclass correlation coefficient for the CNI was 0.85 for the total CNI score. The intraclass correlation coefficients for the subscales were as follows: motor coordination (0.91), sensory integration (0.82), and disinhibition (0.9). Chan and Chen [34] have demonstrated that the CNI is able to discriminate between patients with schizophrenia and healthy controls in the context of Chinese setting, using the three subscales of neurological soft signs.

### Statistical Analysis

SEM was conducted with the LISREL 8.53 for Windows [49] used to analyze the current findings. SEM is a statistical technique for testing and estimating causal relationships using a combination of statistical data and qualitative causal assumptions. The SEM consists of two models, namely the measurement model and structure model [50]. The measurement model shows the relations between the latent variables and their indicators, whereas the structure model shows the potential causal dependencies between endogenous and exogenous variables. In the current study, the measurement model was based on a four-factor measurement model consisting of four latent variables, namely the Executive Functions, Verbal Memory, Visual Memory, and Neurological Soft Signs. Specifically, the SART Correct Response, Verbal Fluency, and WCST Category were ascribed to “Executive Functions”; the Logical Memory Immediate Recall and Logical Memory Delayed Recall were ascribed to “Verbal Memory”; and the Visual Reproduction Immediate Recall and Visual Reproduction Delayed Recall were ascribed to “Visual Memory”. On the other hand, the Motor Coordination, Sensory Integration, and Disinhibition were ascribed to “Neurological Soft Sign”. For the structural model, it shows the relationships between the conventional neurocognitive functions, i.e., Executive Functions, Verbal Memory, and Visual Memory, and the latent variable of Neurological Soft Signs (which was contributed by the motor coordination, sensory integration, and disinhibition).

The validity of the model was tested with chi-square test and fit indices. Five fit indices have been developed for evaluating how the model fits the data, namely the Normed Fit Index (NFI), Non-Normed Fit Index (NNFI), Comparative Fit Index (CFI), Incremental Fit Index (IFI), and RMSEA (Root Mean Square Error of Approximation). These indices represent the improvement in fit between the assumed model and the baseline model of uncorrelatedness between the observed variables. The first four fit indices values of .90 or above, and RMSEA value of .08 or less indicated the model adequately fits the data [51], [52]. Moreover, in checking the validity of the model, we conducted the SEM analysis separately for the patients with schizophrenia and healthy volunteers.

### Procedures

This study was approved by the ethics committees of the corresponding hospitals and the Institute of Psychology, Chinese Academy of Sciences, the Institute of Mental Health of Peking University, and the University of Hong Kong. Informed written consent was obtained from all participants before the testing session according to the Declaration of Helsinki. A trained research assistant administered the tests in a quiet cubicle.

## Results

### Preliminary Analyses

Means, standard deviation, and zero-order correlation among the performance of 11 tests of 209 participants are shown in Table 1. For further analyses, the raw scores of each neurocognitive test of sample were transformed to standardized scores (mean of 0, SD of 1, range −3 to3).

**Table 1:** Means, Standard Deviation, and Zero-Order Correlation among Performance of 10 Tests in Patients with Schizophrenia and Healthy Volunteers.

Tests	M	SD	1	2	3	4	5	6	7	8	9	10
Sch Patient Sample (N = 118)												
SART Correct Response	165.58	37.57	–	.51**	−.42**	.38**	.34**	.49**	.49**	−.33**	−.25**	−.02
Verbal Fluency	14.17	6.23		–	−.42**	.48**	.46**	.63**	.55**	−.37**	−.41**	−.05
WCST Perseverative Error	14.47	14.91			–	−.43**	−.45**	−.34**	−.35**	.33**	.22**	−.10
Logical Memory Immediate Recall	6.28	4.32				–	.83**	.53**	.54**	−.38**	−.38**	−.04
Logical Memory Delayed Recall	4.20	4.03					–	.49**	.47**	−.38**	−.31**	−.01
Visual Reproduction Immediate Recall	15.92	6.99						–	.84**	−.44**	−.41**	−.11
Visual Reproduction Delayed Recall	13.48	7.90							–	−.42**	−.38**	.00
Motor Coordination	4.44	2.83								–	.61**	.41**
Sensory Integration	2.00	1.72									–	.34**
Disinhibition	3.30	1.74										–
Healthy Sample (N = 160)												
SART Correct Response	196.44	7.24	–	.30**	−.48**	.33**	.32**	.31**	.32**	−.30**	−.20*	−.22**
Verbal Fluency	21.71	6.10		–	−.31**	.31**	.29**	.41**	.43**	−.14	−.24**	−.02
WCST Perseverative Error	1.63	3.01			–	−.32**	−.30**	−.26**	−.28**	.20*	.20*	.13
Logical Memory Immediate Recall	14.66	4.02				–	.88**	.26**	.31**	−.23**	−.05	−.11
Logical Memory Delayed Recall	12.82	4.48					–	.23**	.30**	−.24**	−.07	−.10
Visual Reproduction Immediate Recall	22.93	2.13						–	.76**	−.02	−.20*	−.20*
Visual Reproduction Delayed Recall	22.66	2.12							–	.02	−.22**	−.06
Motor Coordination	1.21	1.62								–	.37**	.32**
Sensory Integration	1.18	1.50									–	.31**
Disinhibition	1.22	1.06										–

Note. * p<.05, ** p<.01.

1: SART correct response; 2: Verbal Fluency; 3: MCST perseverative error; 4: Logical Memory Immediate Recall; 5: Logical Memory Delayed Recall; 6: Visual Reproduction Immediate Recall; 7: Visual Reproduction Delayed Recall; 8: Motor Coordination; 9: Sensory Integration; 10: Disinhibition.

Cutoff of correlation coefficient at 0.05 level is 0.197 and at 0.01 level is 0.256 for df = 100; cutoff of correlation coefficient at 0.05 level is 0.139 and at 0.01 level is 0.182 for df = 200.

### Testing the Measurement Model

The results showed that the four-factor measurement model fit the data relatively well. All of the loadings of the observed variables on corresponding latent variables were above 0.4 and statistically significant (p<0.01, see Table 2). Thus, all of the latent variables appear to have been adequately measured by their respective observed variables. Furthermore, correlations between the independent latent variable (Soft Sign) and dependent latent variable (i.e. Attention/Executive Function, Logical Memory, and Visual Memory) were all statistically significant (p<0.01, see Table 3).

**Table 2:** Factor Loadings for the Measurement Model in Patients with Schizophrenia and Healthy Volunteers.

Measure and Variable	Sch Patient Sample (N = 118)				Healthy Sample (N = 160)			
	Untandardized factor loading	SE	Standardized factor loading	p	Untandardized factor loading	SE	Standardized factor loading	p
**Attention/Executive Function**								
SART Correct Response	24.54	3.38	.65	.000	4.77	.61	.66	.000
Verbal Fluency	4.92	.54	.79	.000	3.39	.52	.56	.000
WCST Perseverative Error	−8.30	1.39	−.56	.000	−1.83	.25	−.61	.000
**Logical Memory**								
Logical Memory Immediate Recall	4.07	.33	.94	.000	3.85	.28	.96	.000
Logical Memory Delayed Recall	3.55	.32	.88	.000	4.12	.32	.92	.000
**Visual Memory**								
Visual Reproduction Immediate Recall	6.60	.51	.94	.000	1.78	.17	.84	.000
Visual Reproduction Delayed Recall	7.03	.60	.89	.000	1.93	.17	.91	.000
**Soft Sign**								
Motor Coordination	2.44	.26	.86	.000	1.10	.17	.68	.000
Sensory Integration	1.25	.16	.73	.000	.84	.15	.56	.000
Disinhibition	.75	.17	.43	.000	.51	.10	.48	.000

**Table 3:** Correlations among Latent Variables for the Measurement Model in Patients with Schizophrenia and Healthy Volunteers.

Latent variable	Executive Attention	Verbal Memory	Visual Memory	Neurological Soft Sign
Sch Patient Sample (N = 118)				
Executive Attention	–	.67**	.80**	−.56**
Verbal Memory		–	.60**	−.47**
Visual Memory			–	−.54**
Neurological Soft Sign				–
Healthy Sample (N = 160)				
Executive Attention	–	.54**	.61**	−.54**
Verbal Memory		–	.34**	−.26**
Visual Memory			–	−.17*
Neurological Soft Sign				–

Note. * p<.05, ** p<.01.

Cutoff of correlation coefficient at 0.05 level is 0.197 and at 0.01 level is 0.256 for df = 100; cutoff of correlation coefficient at 0.05 level is 0.139 and at 0.01 level is 0.182 for df = 200.

### Testing the Structure Model

**Figure 1:**
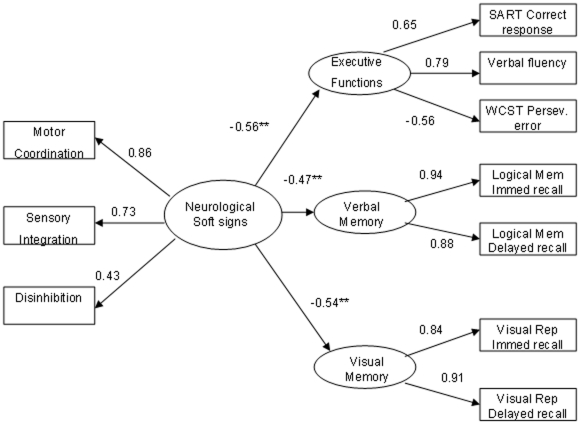
The structure model of the influence of Soft Sign on cognitive function in patients with schizophrenia. χ^2^ (29) = 39.48, p = 0.093, NFI = 0.96, NNFI = 0.98, CFI = 0.99, IFI = .99, RMSEA = 0.056. Note. * p<0.05, ** p<0.01.

**Figure 2:**
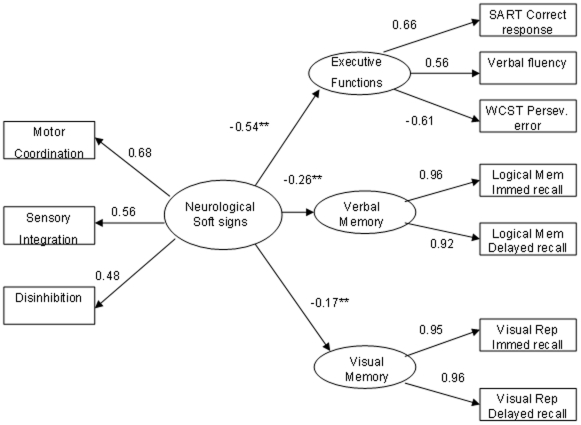
The structure model of the influence of Soft Sign on cognitive function in healthy controls. χ^2^ (29) = 47.79, p = 0.015, NFI = 0.93, NNFI = 0.95, CFI = 0.97, IFI = 0.97, RMSEA = 0.064. Note. * p<0.05, ** p<0.01.

The results showed a good fit of the structure model to the data in both samples. In the patient group, the model fit well to the data, χ2 (29) = 39.48, p = 0.093, NFI = 0.96, NNFI = 0.98, CFI = 0.99, IFI = . 099, RMSEA = 0.056. The structural paths from neurological soft signs to executive attention, verbal memory, and visual memory were −0.56, −0.47, and −0.54, respectively, and all statistically significant (p<0.01, see Figure 1).In the control group, similar fitting was demonstrated, χ^2^ (29) = 47.79, p = 0. 0015, NFI = 0.93, NNFI = 0.95, CFI = 0.97, IFI = 0.97, RMSEA = 0.064. The structural paths from neurological soft signs to executive attention, verbal memory, and visual memory were −0.54, −0.26, and −0.17, respectively, and all statistically significant (p<0.01, see Figure 2). These results suggest that neurological soft signs have important negative influence on executive attention, verbal memory, and visual memory. In other words, greater evidence of neurological soft signs is associated with more severe impairment of executive attention and memory functions.

## Discussion

The current findings indicate that, on the one hand, in the measurement model of the SEM that motor coordination, sensory integration, and disinhibition subscales contributed to the latent construct of neurological soft signs, whereas the subset of neurocognitive function tests contributed to the latent constructs of executive attention, verbal memory, and visual memory in the present sample. On the other hand, the structural model showed that there were significant structural pathways from neurological soft signs to executive attention, verbal memory, and visual memory. In other words, more evidence of neurological soft signs is associated with more severe impairments in executive attention and memory functions.

To a large extent, these findings are consistent with previous correlational studies, namely that neurological soft signs are associated with specific neurocognitive deficits rather than a generalized dysfunction [15], [16], [22]. Instead of linking different subscales of neurological soft signs to specific domains of neurocognitive functions, the main uniqueness of the current findings is the use of the SEM to translate the composition of the soft signs categories into a latent construct of neurological soft signs and to link these signs to specific neurocognitive deficits. As we argue above, the strength of this approach is to examine these two constructs (neurological soft signs and neurocognitive functions) at an explanatory level rather than an exploratory level.

The current findings tend to support the view that neurological soft signs and neurocognitive tests are two ways of capturing the same construct, i.e., ultimate brain functioning. Most recent empirical findings from structural [29], [53] and functional imaging [24], [54]–[56] also suggest that specific brain structural changes reflect a common neuroanatomical substrate of neurological soft signs, across patients with schizophrenia and non-clinical subjects. For example, Schroder et al. [56] found that there were significant reductions in brain activation in the sensorimotor cortex and supplementary motor areas in schizophrenia as compared to healthy controls while they were performing a diadochokinesia like task (pronation/supination). Chan et al. [24] and Rao et al. [54] further showed that the involvement of the supplementary motor area might be the genesis of the Fist-Edge-Palm sign (one of the motor coordination signs) in healthy volunteers and there was a reduction of brain activation in the network between right inferior and right middle prefrontal areas when healthy volunteers were instructed to perform such a task. The data from the patients and healthy volunteers provide converging evidence of the current models.

The regression coefficients between neurological soft signs and the conventional neurocognitive functions were more significant and greater in the patient group than the healthy volunteers group. This is consistent with the *a priori hypothesis* that similar but attenuated associations would be found between conventional neurocognitive functions and neurological soft signs in healthy volunteers. However, it should be noted that while the measurement models of these two samples were meaningful, the p-values for the structural model of schizophrenia and healthy volunteers are 0.96 and 0.015, respectively. That means the data in the schizophrenia sample were more stable and stringent in reflecting the underlying relationships between these constructs (neurological soft signs and conventional neurocognitive functions) than those in healthy volunteers. This might be due to the fact that the prevalence rate of neurological soft signs in healthy volunteers is much lower than that of schizophrenic patients and most of the healthy volunteers might not suffer from any deficits of neurocognitive functions.

Despite the rigorous adoption of SEM, the current study is limited by several methodological design features. Further work is warranted to address the concept that neurological soft signs and neurocognitive functions are measuring the same construct. First, the patients and healthy controls were not well-matched demographically. Although the original purpose of the current study was not to test the group differences, we would like to comment about the similarity or difference in the patterns of associations in the neurocognitive performances and neurological soft signs evidence. Age and IQ may have influenced these associations. However, IQ is influenced by the illness and thus might not be controlled, but age and neurological soft signs might be linked to each other [22], [28]. The current findings are limited by the lack of psychological assessment of IQ in the schizophrenia sample, and therefore, the impossibility of testing for differences in IQ between clinical and non-clinical samples. Nevertheless, a similar but attenuated pattern of associations between neurocognitive functions and neurological soft signs was also demonstrated in healthy controls. Further study with a demographically matched control group to demonstrate a clearer association between these two supposedly similar constructs could address this concern.

Second, the patient sample was biased to chronic cases with relatively long lengths of illness and relatively large medication exposures. It is not clear whether the relationships found in the chronic cases may be generalized to first-onset medication naïve patients. However, previous correlational studies on medication naïve patients suggest that there is quite a robust relationship between neurological soft signs and neurocognitive impairments and medication has little effect on these relationships ([5], [19] for review). Also, both chronic and first-episode medication treated cases suffer similar neurocognitive impairments but the medication naïve cases demonstrate a lesser extent of impairments [57], [58].

Third, the study adopted the subscales of CNI for the evaluation of neurological soft signs, which is different from the Neurological Evaluation Scale (NES) [5] often used in previous studies. The two scales share commonalities in their subscales such as the inclusion of items from the sensory integration and motor coordination. However, the CNI differs from the NES in that it incorporates the complex sequencing task items into its motor coordination subscale instead of classifying it as an independent subscale of complex motor sequencing, whereas the NES categorizes some of the signs of CNI into “other signs”. On the other hand, it includes items that capture the ability to inhibit involuntary and associated movements such as head movement while performing a saccadic task and mirror (associated) movements observed in the un-tested hand. Once again, it is not sure whether similar relationship could be generalized to a different classification of neurological soft signs in schizophrenia. Nevertheless, studies adopting either the CNI [6], [22], [28], [34], [38], [45] or NES [4], [7], [15], [17], [19] have shown impressive sensitivity and discriminating power as a comprehensive neurological soft signs battery, and thus, suggest a valid classification of either approach. More importantly, the same structural and measurement models derived from the assessment of both patients and healthy volunteers provide strong support for the validity of the current observations.

Finally, the current findings were limited to Han Chinese. Given the potential ethnicity effect on the prevalence rate of neurological soft signs in either healthy volunteers or schizophrenic patients, it is not clear such findings can be generalized to the western-based samples, which are the main trend of findings published in the current literature.

Notwithstanding these caveats, the current findings provide one of the very few preliminary supports for the claim that neurological soft signs and conventional neurocognitive functions may measure at the same level of brain functions. These findings may add knowledge to the understanding of the neural basis of these apparently distinct constructs is actually capturing the same or similar neural basis of higher cortical functioning in schizophrenia. The significant association of these two constructs, particularly for those neurocognitive functions regarding as endophenotypes such as verbal memory and executive functions, may imply that neurological soft signs may also serve as the potential cognitive endophenotypes for schizophrenia [4], [19], [20]. The clinical implication could l be straightforward, i.e. administration of neurological soft signs is simpler and time-saving for the already tight daily clinical routines and the rating of these signs are portable and user-friendly as an alternative to conventional neurocognitive function assessment for schizophrenia or related disorders research in the future.

## References

[1] Kennard MA (1960). Value of equivocal signs in neurologic diagnosis.. Neurology.

[2] Rossi A, de Cataldo S, di Michele V, Manna V, Ceccoli S (1990). Neurological soft signs in schizophrenia.. Br J Psychiatry.

[3] Gupta S, Andreasen NC, Amdt S, Flaum M, Schultz SK (1995). Neurological soft signs in neuroleptic-naïve and neuroleptic-treated schizophrenic patients and in normal comparison subjects.. Am J Psychiatry.

[4] Heinrichs DW, Buchanan RW (1988). Significance and meaning of neurological signs in schizophrenia.. Am J Psychiatry.

[5] Buchanan RW, Heinrichs DW (1989). The Neurological Evaluation Scale (NES): a structured instrument for the assessment of neurological signs in schizophrenia.. Psychiatry Res.

[6] Chen EYH, Shapleske J, Luque R, McKenna PJ, Hodges JR (1995). The Cambridge Neurological Inventory: a clinical instrument for soft neurological signs and the further neurological examination for psychiatric patients.. Psychiatry Res.

[7] Candela S, Manschreck T (2003). Neurologic signs in schizophrenia: Research findings and clinical relevance.. Psychiatr Ann.

[8] Fish B (1977). Neurobiologic antecedents of schizophrenia in children.. Arch Gen Psychiatry.

[9] Marcus J, Hans SL, Lewow E, Wilkinson L, Burack M (1985). Neurological findings in high-risk children: childhood assessment and 5-year follow up.. Schizophr Bull.

[10] Lawrie SM, Byrne M, Miller P, Hodges A, Clafferty RA (2001). Neurodevelopmental indices and the development of psychotic symptoms in subjects at high risk of schizophrenia.. Br J Psychiatry.

[11] Tsuang MT, Gilberson MW, Faraone SV (1991). The genetics of schizophrenia: Current knowledge and future directions.. Schizophr Res.

[12] Tsuang MT, Faraone SV (1999). The concept of target features in schizophrenia research.. Acta Psychiatr Scand Supplement.

[13] Heinrichs RW, Zakzanis KK (1998). Neurocognitive deficit in schizophrenia: A quantitative review of the evidence.. Neuropsychology.

[14] Heinrichs DW (2005). The primacy of cognition in schizophrenia.. Am Psychol.

[15] Flashman LA, Flaum M, Gupta S, Andreasen NC (1996). Soft signs and neuropsychological performance in schizophrenia.. Am J Psychiatry.

[16] Wong AHC, Voruganti LNP, Heslegrave RJ (1997). Neurocognitive deficits and neurological signs in schizophrenia.. Schizophr Res.

[17] Arango C, Bartko JJ, Gold JM, Buchanan RW (1999). Prediction of neuropsychological performance by neurological signs in schizophrenia.. Am J Psychiatry.

[18] Chan RCK, Toulopoulou T, Columbus F (2006). Fractionation of executive function in schizophrenia: relationships to clinical and neurological manifestations.. Schizophrenic psychology: New research.

[19] Bombin I, Arango C, Buchanan RW (2005). Significance and meaning of neurological signs in schizophrenia: Two decades later.. Schizophr Bull.

[20] Chan RCK, Gottesman II (2008). Neurological soft signs as candidate endophenotypes for schizophrenia: A shooting star or a Northern star?. Neurosci Biobehav Rev.

[21] Chen EYH, Lam LCW, Chen RYL, Nguyen DGH (1996). Negative symptoms, neurological signs and neuropsychological impairments in 204 Hong Kong Chinese patients with schizophrenia.. Br J Psychiatry.

[22] Chen EYH, Lam LCW, Chen RYL, Nguyen DGH, Kwok CL (2001). Neurological signs and sustained attention impairment in schizophrenia.. Eur Arch Psychiatry Clin Neurosci.

[23] Chan RCK, Chen EYH (2004). Blink rate does matter: a study of sustained attention and neurological signs in schizophrenia.. J Nerv Ment Dis.

[24] Chan RCK, Rao HY, Chen EYH, Ye BB, Zhang C (2006). The neural basis of motor coordination soft signs: an fMRI study of healthy subjects.. Neurosci Lett.

[25] Mohr F, Hubmann W, Albus M, Franz U, Hecht S (2003). Neurological soft signs and neuropsychological performance in patients with first episode schizophrenia.. Psychiatry Res.

[26] Mohr F, Hubmann W, Bender W, Honicke S, Wahlheim C (1996). Neurological soft signs in schizophrenia: assessment and correlates.. Eur Arch Psychiatry Clin Neurosci.

[27] Gureje O (1988). Neurological soft signs in Nigerian schizophrenics: A controlled study.. Acta Psychiatr Scand.

[28] Chen EYH, Chan RCK (2003). The Cambridge Neurological Inventory: Clinical, demographic and ethnic correlates.. Psychiatr Ann.

[29] Dazzan P, Morgan KD, Chitnis X, Suckling J, Morgan C (2006). The structural brain correlates of neurological soft signs in healthy individuals.. Cereb Cortex.

[30] Kinney DK, Woods BT, Yurgelun-Todd MA (1986). Neurological abnormalities in schizophrenic patients and their families.. Arch Gen Psychiatry.

[31] Griffiths TD, Sigmundsson T, Takei N, Rower D, Murray RM (1998). Neurological abnormalities in familial and sporadic schizophrenia.. Brain.

[32] Niethammer R, Weisbrod M, Schiesser S, Grothe J, Maier S (2000). Genetic influence on laterality in schizophrenia? A twin study of neurological soft signs.. Am J Psychiatry.

[33] Yazici AH, Demir B, Yazici KM, Gogus A (2002). Neurolgoical soft signs in schizophrenic patients and their nonpsychotic siblings.. Schizophr Res.

[34] Chan RCK, Chen EYH (2007). Neurological Abnormalities in Chinese patients with schizophrenia.. Behav Neurol.

[35] Spikeman JM, Kiers HAL, Deelman BG, van Zomeren AH (2001). Construct validity of concepts of attention in healthy controls and patients with CHI.. Brain Cogn.

[36] Gottesman II, Shields J (1973). Genetics theorizing and schizophrenia.. Br J Psychiatry.

[37] Luria AR (1966). Higher cortical functions in man.

[38] Chan RCK, Chen EYH, Cheung EFC, Cheung HK (2004). Executive dysfunctions in schizophrenia: relationships to clinical manifestations.. Eur Arch Psychiatry Clin Neurosci.

[39] Kaufman A, Kaufman N (1983). Assessment Battery for Children.

[40] Korkman M (1999). Applying Luria's diagnostic principles in the neuropsychological assessment of children.. Neuropsychol Rev.

[41] Fox G, Fox AM (2001). The effects of brain damage on the performance of hand movement sequences.. Brain Impair.

[42] American Psychiatric Association (1994). Diagnostic and Statistical Manual of Mental Disorders. 4th ed.

[43] Gong YX (1992). Manual of Wechsler Adult Intelligence Scale-Chinese version.

[44] Robertson IH, Manly T, Andrade J, Baddeley BT, Yiend J (1997). “Oops!”: Performance correlates of everyday attentional failures in traumatic brain injured and normal subjects.. Neuropsychologia.

[45] Chan RCK, Chen EYH, Cheung EFC, Chen RYL, Cheung HK (2004). A study of sensitivity of the Sustained Attention to Response Task in patients with schizophrenia.. Clin Neuropsychol.

[46] Nelson HE (1976). A modified card sorting task sensitive to frontal lobe defects.. Cortex.

[47] Heaton RK (1993). Wisconsin Card Sorting Test: Computer Version-2..

[48] Gong YX, Gong YX, Jiang DW (1989). Manual of Wechsler Memory Scale-Chinese version.

[49] Scientific Software International (2005). LISREL 8.53.

[50] Anderson JC, Gerbing DW (1988). Structural equation modeling in practice: A review and recommended two-step approach.. Psychol Bull.

[51] Hair JF, Anderson RE, Tatham RL, Black WC (1998). Multivariate data analysis with readings. 5th ed.

[52] Hu L, Bentler PM (1999). Cutoff criteria for fit indexes in covariance structure analysis: Conventional criteria versus new alternatives.. Structural Equation Modeling.

[53] Dazzan P, Morgan KD, Orr KG, Huchinson G, Chitnis X (2004). The structural brain correlates of neurological soft signs in AESOP first-episode psychoses study.. Brain.

[54] Rao H, Di X, Chan RCK, Ding Y, Ye B (2008). A regulation role of the prefrontal cortex in the Fist-Edge-Palm Task: evidence from functional connectivity analysis.. Neuroimage.

[55] Gunther W, Petsch R, Steinberg R, Moser E, Streck P (1991). Brain dysfunction during motor activation and corpus callosum alterations in schizophrenia measured by cerebral blood flow and magnetic resonance imaging.. Biol Psychiatry.

[56] Schroder J, Essig M, Baudendistel K, Jahn T, Gerdsen I (1999). Motor dysfunction and sensorimotor cortex activation changes in schizophrenia: a study with functional magnetic resonance imaging.. Neuroimage.

[57] Chan RCK, Chen EYH, Cheung EFC, Chen RYL, Cheung HK (2006). The components of executive functioning in a cohort of patients with chronic schizophrenia: a multiple single-case study design.. Schizophr Res.

[58] Chan RCK, Chen EYH, Law CW (2006). Specific executive dysfunction in patients with first-episode medication-naïve schizophrenia.. Schizophr Res.

